# Single-agent therapy with sorafenib or 5-FU is equally effective in human colorectal cancer xenograft—no benefit of combination therapy

**DOI:** 10.1007/s00384-012-1551-2

**Published:** 2012-09-15

**Authors:** Thomas C. Wehler, Swaantje Hamdi, Annett Maderer, Claudine Graf, Ines Gockel, Irene Schmidtmann, Michael Hainz, Martin R. Berger, Matthias Theobald, Peter R. Galle, Markus Moehler, Carl C. Schimanski

**Affiliations:** 1 Third Department of Internal Medicine, Johannes Gutenberg University Hospital of Mainz, Mainz, Germany; 2 First Department of Internal Medicine, Johannes Gutenberg University Hospital of Mainz, Langenbeckstrasse 1, 55101 Mainz, Germany; 3 Department of General and Abdominal Surgery, Johannes Gutenberg University Hospital of Mainz, Mainz, Germany; 4 Institute of Medical Biostatistics, Epidemiology and Informatics (IMBEI), Johannes Gutenberg University Hospital of Mainz, Mainz, Germany; 5 Institute of Pathology, Johannes Gutenberg University Hospital of Mainz, Mainz, Germany; 6 Toxicology and Chemotherapy Unit, German Cancer Research Center (DKFZ), Im Neuenheimer Feld 581, 69120 Heidelberg, Germany; 7 Department of Internal Medicine, Marienhospital, 64285 Darmstadt, Germany

**Keywords:** Colorectal cancer, TKI, Sorafenib

## Abstract

**Background:**

We initiated this preclinical study in order to analyze the impact of sorafenib single treatment versus combination treatment in human colorectal cancer.

**Methods:**

The effect of increasing sorafenib doses on proliferation, apoptosis, migration, and activation of signal cascades was analyzed in vitro. The effect of sorafenib single treatment versus 5-fluorouracil (5-FU) single treatment and combination therapy on in vivo proliferation and target cytokine receptor/ligand expression was analyzed in a human colon cancer xenograft mouse model using HT29 tumor cells.

**Results:**

In vitro, SW480 and HT29 cell lines were sensitive to sorafenib, as compared to Caco2 and SW620 cell lines, independent of the mutation status of *K-ras*, *Raf*, *PTEN*, or *PI3K*. The effect on migration was marginal, but distinct differences in caspases activation were seen. Combination strategies were beneficial in some settings (sorafenib + 5-FU; irinotecan) and disadvantageous in others (sorafenib + oxaliplatin), depending on the chemotherapeutic drug and cell line chosen. Sensitive cell lines revealed a downregulation of *AKT* and had a weak expression level of *GADD45β*. In resistant cell lines, *pp53* and *GADD45β* levels decreased upon sorafenib exposure. In vivo, the combination treatment of sorafenib and 5-FU was equally effective as the respective monotherapy concerning tumor proliferation. Interestingly, treatment with either sorafenib or 5-FU resulted in a significant decrease of VEGFR1 and PDGFRβ expression intensity.

**Conclusions:**

In colorectal cancer, a sensitivity towards sorafenib exists, which seems similarly effective as a 5-FU monotherapy. A combination therapy, in contrast, does not show any additional effect.

## Introduction

Colorectal cancer ranges among the three most frequent malignancies in Western countries [1, 2]. Survival is determined by local recurrence, lymphatic, and hematogenous dissemination [3]. Due to improved therapeutic strategies, the overall survival in stage IV colorectal cancer has increased from 8 months to more than 2 years during the last decade [4–6].

Besides new chemotherapeutic drugs, such as platinum derivates (oxaliplatin) and topoisomerase II inhibitors (irinotecan), the introduction of biologicals targeting tumor neovascularization or growth signaling significantly has improved response and prognosis [4–6].

Specific mutations in tumor-suppressor genes (APC, DCC, p53) and oncogenes (K-ras) occur during the adenoma–carcinoma sequence of colorectal cancer [7–9]. The K-ras mutation status was the first key to personalized therapy in colorectal cancer, as anti-EGFR strategies were shown to be effective in K-ras wild types only [10].

Receptor tyrosine kinases (RTKs) are transmembrane receptors containing extracellular ligand-binding domains connected to intracellular catalytic domains [11]. The growth factors VEGF/PDGF/EGF and their receptors VEGFR1-3, PDGFRα/β, and EGFR are critical in the process of (lymphatic) neo-angiogenesis and dissemination in human cancer [12–16]. Inhibition of RTKs with sorafenib has been successful in renal and hepatocellular cancers [17, 18]. Two phase I studies revealed a disease stabilization in pretreated colorectal cancer patients receiving sorafenib in combination with either irinotecan or oxaliplatin [19, 20]. However, recent phase II/III studies testing other multi-tyrosine kinase inhibitors in human colorectal cancer have failed to show any benefit [21]. So far only one randomized Phase III study with Regorafenib improved survival times after failure of all approved standard therapies [22]. Therefore, the impact of combinational therapies (sorafenib + chemotherapy) remains controversial. Preclinical data as well as experimental data explaining interaction mechanisms are widely missing. Thus, we initiated this study to examine sorafenib targeted RTK expression and to analyze the in vivo effect of sorafenib alone or in combination with the classical chemotherapeutic backbone 5-fluorouracil (5-FU).

## Material and methods

### Cell culture

The human colorectal cancer cell lines SW480 [K-ras mt, B-Raf wt, PI3K wt, p53 mt], SW620 [K-ras mt, B-raf wt, PI3K wt, p53 mt], and HT29 [K-ras wt, B-raf mt, PI3K wt, p53 mt] were cultured in RPMI 1640 (Invitrogen, Germany) supplemented with 10 % FCS, 100 U/ml penicillin, 100 μg/ml streptomycin (Cambrex, Germany), and 1 mM l-glutamine (Invitrogen, Germany). The human colorectal cancer cell line Caco2 [K-ras wt, B-Raf wt, PI3K wt, p53 mt] was cultured in DMEM (Invitrogen, Germany) supplemented with 10 % FCS, 100 U/ml penicillin, 100 μg/ml streptomycin (Cambrex, Germany), and 1 mM l-glutamine (Invitrogen, Germany).

### Proliferation assays and chemosensitvity

For proliferation assays, 5 × 10^3^ SW480, SW620, Caco2, or HT-29 cells were plated in 96-well plates and cultured as described above. Twelve hours after plating sorafenib (0, 5, and 10 μg/ml), 5-FU (0.5 mg/ml) ± sorafenib (5 μg/ml), irinotecan (1 mg/ml) ± sorafenib (5 μg/ml), or oxaliplatin (0.5 mg/ml) ± sorafenib (5 μg/ml) were added to the medium. The amount of cells per well was determined by luminescence assay (CellTiter-Glo Cell Viability assay, Promega, USA). Each condition was performed in quadruplicates.

For apoptosis analyses, 2 × 10^5^ cells were seeded per 6 wells, respectively. Twelve hours after plating, cells were treated for 24 h as mentioned above. Suspended cells were collected, and adherent cells were trypsinized prior to fixation with 100 % ethanol, staining with propidium iodide and analyzation by FACS without gating. Each condition was performed in quadruplicates.

### Migration assay

SW480, SW620, Caco2, or HT29 cells (2 × 10^6^ ) were seeded per 6 wells, cultured for 24 h, serum-starved (2 % FCS only) for 12 h, and exposed to sorafenib at different concentrations (0, 5, or 10 μg/ml) for 6 h. Migration was assayed with 24-well HTS FluoroBlock Inserts in triplet approaches (8 μM pore size; Becton Dickinson, USA).

In brief, 4×10^4^ cells were resuspended in RPMI1640/DMEM medium containing 2 % FCS and 10 ng/ml CXCL12 and added to the upper chamber. Subsequently, RPMI1640/DMEM medium with 20 % FCS and 100 ng/ml CXCL12 (Sigma, Germany) was added to the lower chamber. Chambers were incubated for 24 h at 37 °C in a humid atmosphere of 5 % CO_2_. After incubation, the amount of migrated cells in the lower chamber was determined by luminescence assay (CellTiter-Glo, Cell Viability assay, Promega, USA). Each condition was performed in triplicate.

### Caspase assay

Cells were treated with placebo or sorafenib (5 and 10 μg/ml, respectively). After incubation for 16 h, cells were lysed in buffer containing 20 mM Tris/HCl pH 8.0, 5 mM EDTA, 0.5 % Triton X-100, and onefold complete protease inhibitor cocktail (Roche, Germany). Protein concentration was determined by Bradford assay (Sigma, Germany). Sixty micrograms of protein was incubated in reaction buffer (25 mM HEPES pH 7.5, 50 mM NaCl, 10 % glycerol, 0.05 % CHAPS, and 5 mM DTT) in the presence of 50 μM fluorogenic substrate (Biomol, Germany), which was specific for caspase 3 (DEVD-AMC); caspase 6, 8, and 10 (Ac-IETD-AFC); or caspase 9 (Ac-LEHD-AFC). Analyses were performed in triplicates.

Assays were performed in black micro-titer plates (Nunc, Germany), and after 1 h incubation at 37 °C, the generation of free AMC or AFC was measured using a fluorometer plate reader (Appliscan, Thermo Fisher, Germany) at an excitation wavelength of 380 nm (AMC and AFC) and an emission wavelength of 460 nm (AMC) or 505 nm (AFC).

### Western blot analysis

SW480, SW620, Caco2, or HT29 cells (2 × 10^6^) were harvested after a 12-h-long exposition to placebo or sorafenib (5 and 10 μg/ml, respectively). Cells were washed twice with phosphate-buffered saline (PBS; 1×) and lysed in 2× RIPA solution. For Western blot analysis, 100 μg of protein was loaded on 8–12 % SDS-PAGE gels, respectively. After separation, the gel was transferred to a PVDF membrane (Roth, Karlsruhe, Germany). Proteins (AKT/pAKT, MEK/pMEK, PI3K/pPI3K, mTOR/pmTOR, P53/pp53, FoxO3a/pFoxO3a, GADD45β, and alpha tubulin) were detected with specific primary antibodies (Table 1; 4 °C, overnight). The specific secondary antibodies were exposed for 1 h at room temperature (Table 1). For visualisation, the Roti Lumin systems 1 and 2 were applied (P79 and P80, Roth, Karlsruhe, Germany). Each condition was performed in duplicates.

**Table 1:** Antibodies used for Western blotting

Antibody	Manufacturer	Order number	Secondary antibody	Size (kDa)	Dilution
Rabbit-anti-human pPI3K	Cell Signaling	4228	Goat-anti-rabbit IgG	85/60	1:1,000
Rabbit-anti-human PI3K	Cell Signaling	4257	Goat-anti-rabbit IgG	85	1:1,000
Rabbit-anti-human pAKT	Cell Signaling	9267	Goat-anti-rabbit IgG	60	1:1,000
Rabbit-anti-human AKT	Cell Signaling	4685	Goat-anti-rabbit IgG	60	1:1,000
Rabbit-anti-human pmTOR	Cell Signaling	2971	Goat-anti-rabbit IgG	289	1:1,000
Rabbit-anti-human mTOR	Cell Signaling	2983	Goat-anti-rabbit IgG	289	1:1,000
Rabbit-anti-human pMEK	Cell Signaling	9121	Goat-anti-rabbit IgG	45	1:1,000
Rabbit-anti-human MEK	Cell Signaling	9122	Goat-anti-rabbit IgG	45	1:1,000
Goat-anti-human GADD45β	Santa Cruz Biotechnology	sc-8776	Donkey-anti-goat IgG	18	1:500
Mouse-anti-human α-Tubulin	Sigma Aldrich	t5168	Goat-anti-mouse IgG	48,5	1:2,000
Goat-anti-mouse IgG	Santa Cruz Biotechnology	sc-2031	–	–	1:10,000
Goat-anti-rabbit IgG	Santa Cruz Biotechnology	sc-2030	–	–	1:10,000
Donkey-anti-goat IgG	Santa Cruz Biotechnology	sc-2033	–	–	1:10,000

### Subcutaneous xenograft tumor system

HT29 tumor cells (1 × 10^7^) were suspended in 0.2 ml pure RPMI1640 medium and 1× PBS (1:1) and applied by subcutaneous injection into the left flank of 7–8-week-old female nod-SCID mice. Nod-SCID mice were irradiated with 1.8 Gy 1 day prior to s.c. injection of tumor cells. As soon as the tumors reached a size of 10 mm, animals received i.p. injections of placebo (group 1; 200 μl, 5 days/week; 25 % cremophor in NaCl 0,9 %), sorafenib (group 2; 200 μl; 5 days per week; 0.12 mg/dose solved in 25 % cremophor; 30 mg/kg/week), 5-FU (group 3; 200 μl; three times a week; 0.18 mg/dose solved in 25 % cremophor; 25 mg/kg/week) or sorafenib + 5-FU (group 4; 200 μl; combination of group 2 and 3). The size of tumors was measured manually twice weekly. Tumors grew for 4 weeks. Thereafter, tumor nodules were excised and measured manually with a vernier micrometer.

### Immunohistochemistry

Excised tumors obtained from the experimental animals were paraffin-embedded. After obtaining adequate slides, the tissue samples were screened for Ki-67, PDGFA, VEGFA, VEGFR1, VEGFR2, PDFGRα, and PDGFRβ protein expression by immunohistochemistry. To that purpose, the tissues were deparaffinized, rehydrated, and subsequently incubated with the respective primary antibodies [anti-PDGFRα (sc-338); 1:200, 2 h, Santa Cruz Biotechnology, CA, USA; anti-PDGFRβ (3169), 1:40, 2 h, Cell Signaling Technology, MA, USA; PDGFA (NBP1-19781), 1:100, 2 h, Novus Biologicals, Cambridge, UK; VEGFA (ab46154), 1:200, 2 h, Abcam plc, Cambridge UK; VEGFR1 (RB-9049-R7), 1:50, 2 h, Thermo Fisher Scientific GmbH Neomarkers, Germany; VEGFR2 (RB-9239-R7), 1:50, 2 h, Thermo Fisher Scientific GmbH Neomarkers, Germany; VEGFR3 (sc-321), 1:200, 2 h, Santa Cruz Biotechnology, Germany; Ki-67 (mib1), 1:100, 2 h, Dako, Germany; Envision flex plus^TM^, Autostainer, Dako, Germany]. The secondary antibody (anti-rabbit-mouse-goat antibody) was incubated for 15 min at room temperature, followed by incubation with streptavidin-POD (Dako, Germany) for 15 min. Antibody binding was visualized using AEC solution (Dako, Germany). Afterwards, the tissues were counterstained by haemalaun solution (Dako, Germany). The expression of the respective tyrosine kinase was evaluated using a scoring system. Expression strength of PDGFA, VEGFA, VEGFR1, VEGFR2, VEGFR3, PDFGRα, and PDGFRβ was classified as negative (0), low (1), medium, (2) and high (3). All slides were independently evaluated by three investigators. The Ki-67 expression was measured as percentage of Ki-67 expressing cells.

### Statistics

In order to assess dependence of growth factor and Ki-67 expression on treatment with 5-FU and sorafenib, the minimum, the maximum, the median, and the quartiles in subgroups were calculated. For Ki-67 analyses, the mean and standard deviations were calculated and displayed in box plots. Ki-67 was measured three times for each specimen; averages were analyzed using two-way analysis of variance. To compare growth factor expression between treatment groups the Kruskal–Wallis tests was used, followed by pairwise Wilcoxon test if the Kruskal–Wallis test gave a *p* value ≤0.05.

All tests were performed with exploratory intention, associations with *p* values ≤0.05 might warrant further consideration. Statistical analysis was performed using SAS 9.3 2002–2010 by SAS Institute Inc., Cary, NC, USA.

## Results

### Proliferation assay

**Fig. 1:**
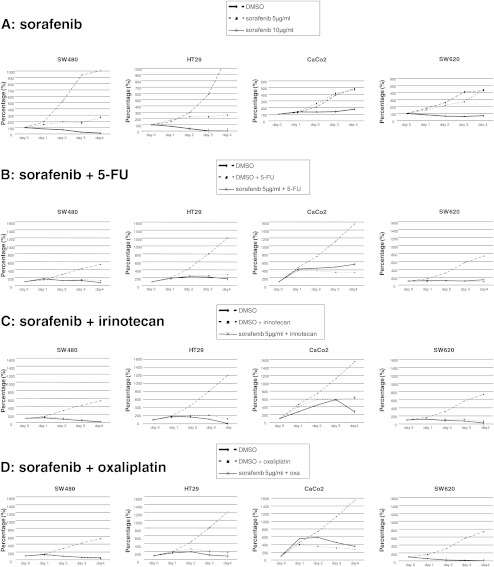
Effect of sorafenib monotherapy and combining sorafenib with standard chemotherapeutics 5-FU, oxalipaltin, or irinotecan. *a* While SW480 and HT29 cells were almost eradicated by high dose sorafenib treatment, low doses resulted in a significantly reduced growth as compared to placebo treatment. In contrast, Caco2 and SW620 cells were resistant to low-dose sorafenib while high-dose sorafenib stabilized tumor cell load of SW620 and Caco2. *b* 5-Fluorouracil**.** Combining 5-FU and sorafenib revealed additive effects (proliferation and apoptosis) in SW480 and HT 29 cells but not in SW620 or Caco2 cells. In contrast, the combination even increased proliferation of Caco2 cells. *c* Irinotecan. Combining 5-FU and irinotecan demonstrated clear additive effects in SW480, HT29 (proliferation and apoptosis), and SW620 (proliferation only) cells and even slightly increased apoptosis of Caco2 cells. However, no effect was seen in SW620 cells. *d* Oxaliplatin. Combining oxalipaltin and sorafenib revealed an additive effect (proliferation and apoptosis) in HT29 cells only but not in SW480, SW620, or Caco2 cells. In contrast, the combination increased proliferation of SW480, Caco2, and SW620 cells and inhibited apoptosis of SW480 and SW620 cells

Inhibition of tumor growth through low dose sorafenib was seen in all cell lines, except for Caco2 (Table 2; Fig. 1a). High-dose sorafenib eradicated SW480 and HT29 cells significantly and SW620 cell less effectively, whereas Caco2 cells revealed a decelerated tumor cell growth only. Mutation status of K-ras, B-Raf, PI3K, or p53 did not correlate with resistance.

**Table 2:**
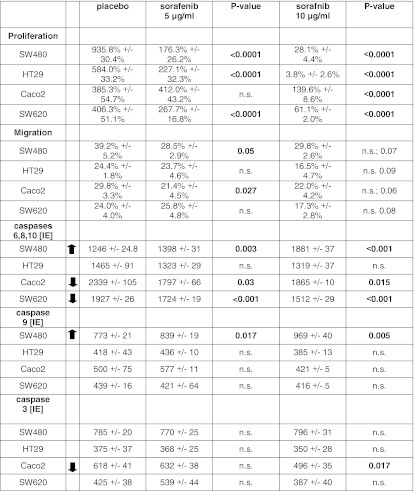
Functional effects of sorafenib in distinct colorectal cancer cell lines

### Migration assay

The treatment with low-dose sorafenib (5 μg/ml) significantly inhibited migration only in SW480 and Caco2 cells (Table 2). High-dose (10 μg/ml) sorafenib nonsignificantly inhibited migration in HT29 and SW620 cells.

### Caspases assays

#### Caspases 6, 8, and 10

Treatment with sorafenib significantly induced caspases 6, 8, and 10 activity in SW480 but not in HT29 cells (Table 2). In contrast, exposure to sorafenib significantly decreased caspases 6, 8, and 10 activity in Caco2 and SW620 cells

#### Caspase 9

Treatment with sorafenib significantly induced caspase 9 activity in SW480 but did not impact on HT29, Caco2, or SW620 cells.

#### Caspase 3

Treatment with sorafenib did not modify caspase 3 activity in SW480, HT29, or SW620 cells. However, caspase 3 activity was significantly decreased in Caco2 cells.

### Chemosensitivity assay (apoptosis and proliferation)

#### 5-Fluorouracil

Combining 5-FU and sorafenib revealed additive effects (proliferation and apoptosis) in SW480 and HT 29 cells but not in SW620 or Caco2 cells (Fig. 1b; Table 3). In contrast, the combination even increased proliferation of Caco2 cells.

**Table 3:** Effect of combining sorafenib with classical chemotherapeutic drugs

		SW480 (%)	*p* value	HT29 (%)	*p* value	Caco2 (%)	*p* value	SW620 (%)	*p* value
Proliferation									
5-FU +	Placebo	150.3 ± 16.0	**0.061**	262.3 ± 25.9	**0.004**	337.2 ± 25.3	**<0.001**	97.2 ± 8.5	0.186
	Sorafenib	127.7 ± 11.9		218.5 ± 20.2		468.2 ± 29.1		104.1 ± 8.5	
Irinotecan +	Placebo	59.0 ± 7.0	**0.002**	217.7 ± 23.8	**<0.001**	585.1 ± 51.9	0.66	120.6 ± 9.7	**<0.001**
	Sorafenib	39.1 ± 2.6		116.2 ± 13.1		570.4 ± 78.6		75.1 ± 8.1	
Oxaliplatin +	Placebo	56.6 ± 3.5	0.1	204.7 ± 36.1	**<0.001**	308.4 ± 33.2	**<0.001**	10.2 ± 0.8	**<0.001**
	Sorafenib	61.7 ± 6.6		114.8 ± 28.6		450.1 ± 20.0		13.7 ± 1.7	
Apoptosis									
5−FU		17.23 ± 2.67	**0.002**	5.28 ± 0.07	**0.047**	14.38 ± 1.15	0.32	5.84 ± 2.45	0.15
5−FU + sorafenib		42.27 ± 0.74		20.55 ± 1.62		16.98 ± 3.38		10.98 ± 1.73	
Sorafenib		16.43 ± 1.45	**<0.001**	11.78 ± 1.56	**0.031**	17.12 ± 0.37	0.95	9.07 ± 4.65	0.66
Irinotecan		41.62 ± 4.12	**0.004**	24.08 ± 2.82	**<0.001**	12.38 ± 2.79	**0.041**	39.07 ± 3.01	0.238
Irinotecan + sorafenib		71.57 ± 1.25		56.23 ± 1.1		19.74 ± 3.2		26.44 ± 7.96	
Sorafenib		16.43 ± 1.45	**<0.001**	11.78 ± 1.56	**0.002**	17.12 ± 0.37	0.29	9.07 ± 4.65	0.146
Oxaliplatin		39.26 ± 1.23	**<0.001**	24.93 ± 1.87	**<0.001**	38.52 ± 3.12	0.6	40.71 ± 1.31	0.058
Oxaliplatin + sorafenib		31.47 ± 1.15		46.92 ± 1.21		36.84 ± 2.13		30.62 ± 0.04	
Sorafenib		16.43 ± 1.45	**<0.001**	11.78 ± 1.56 %	**<0.001**	17.12 ± 0.37	**<0.001**	9.07 ± 4.65	**<0.001**

#### Irinotecan

Combining 5-FU and irinotecan demonstrated clear additive effects in SW480, HT29 (proliferation and apoptosis), and SW620 (proliferation only) cells and even slightly increased apoptosis of Caco2 cells (Fig. 1c). However, no effect was seen in SW620 cells.

#### Oxaliplatin

Combining oxalipaltin and sorafenib revealed an additive effect (proliferation and apoptosis) in HT29 cells only but not in SW480, SW620, or Caco2 cells (Fig. 1d). In contrast, the combination increased proliferation of SW480, Caco2, and SW620 cells and inhibited apoptosis of SW480 and SW620 cells.

### Signal cascade inhibition

**Fig. 2:**
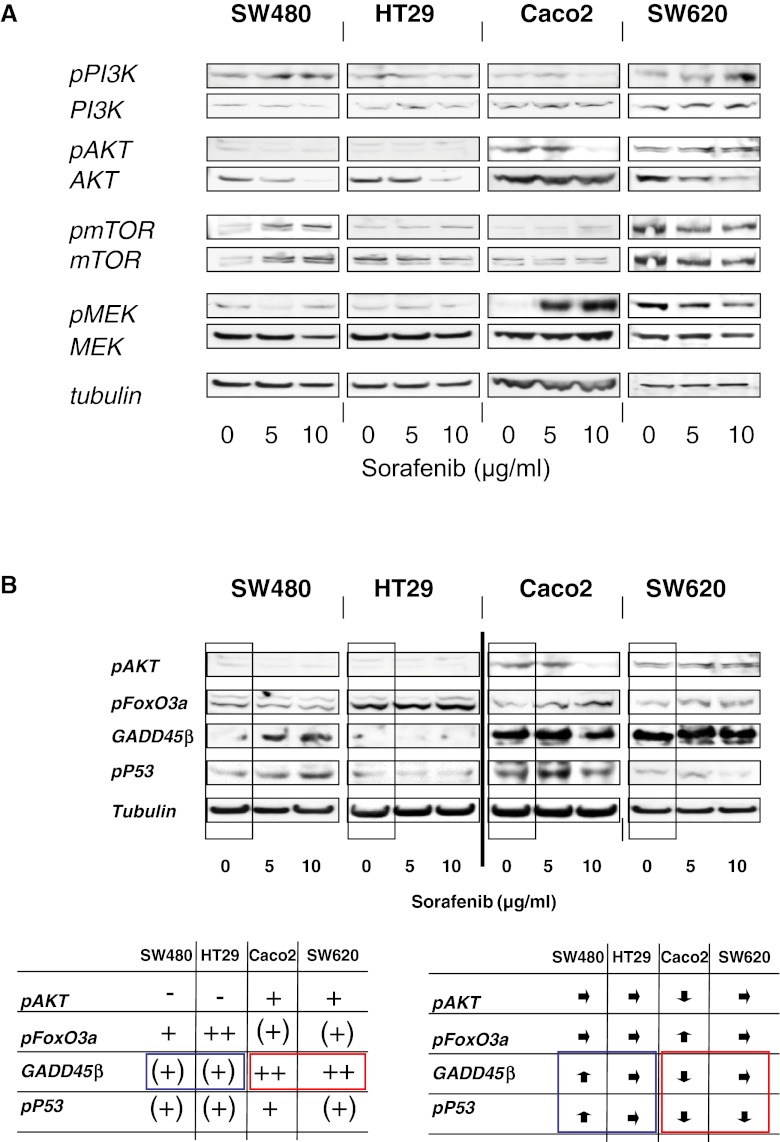
**a** Upon exposure with augmenting sorafenib doses, we observed an inhibition of the Ras–Raf pathway (*pMEK*) in Sw620 cell lines, only. In contrast, this pathway was activated in Caco2 cells. The *AKT* pathway was particularly altered in sensitive cell lines. SW480 and HT29 cells revealed only a hint–absent *pAKT* expression, but *AKT* expression was clearly suppressed upon exposure to increasing sorafenib doses. In contrast, the resistant cell line Caco2 did not show such *AKT* inhibitory behavior. An inhibition of *pPI3K* and *pAKT* was seen in Caco2 but not in SW620. **b** Analyses of the signaling pathways showed that sorafenib-sensitive cell lines reveal almost absent *pAKT*, absent–weak *GADD45β*, and medium–strong *FoxO3a* expression levels. In contrast, resistant cell lines showed a medium *pAKT*, very strong *GADD45β*, and weak–medium *FoxO3a* expression levels. *GADD45β* expression levels discriminated best between sensitive and resistant cell lines. When analyzing the impact of sorafenib, we observed that the sensitive cell line SW480 revealed a *pp53* and a *GADD45ß* upregulation upon exposure to increasing sorafenib doses. In contrast, resistant cell lines showed initially high (Caco2, SW620) and, upon drug exposure, decreasing *GADD45β* (Caco2) and decreasing *pp53* (Caco2, SW620) expression levels

In order to investigate the relevance of sorafenib in the inhibition of signal cascades, we analyzed diverse pathways (Fig. 2a). Upon exposure with increasing sorafenib doses, we observed an inhibition of the Ras–Raf pathway (*pMEK*) in SW620 but an induction in Caco2 cell lines. This pathway remained unchanged in SW480 and HT29 cells.

The *AKT* pathway was specifically altered in sensitive cell lines: SW480 and HT29 cells revealed only weak–absent *pAKT*, but *AKT* expression was significantly suppressed upon exposure with increasing sorafenib doses. In contrast, the resistant cell line Caco2 did not show any *AKT* suppressive behavior. An inhibition of *pPI3K* and *pAKT* was seen in Caco2 but not in SW620.

These results raise the question of whether a suppression of *AKT* expression correlates with responsiveness to sorafenib. As it has been previously reported that *GADD45ß* takes control when *AKT* is absent, we analyzed *GADD 45*-associated genes.

### Potential resistance indicators

We observed that sorafenib-sensitive cell lines revealed almost absent *pAKT*, weak *GADD45β*, and medium–strong *FoxO3a* expression levels (Fig. 2b). In contrast, resistant cell lines showed medium *pAKT*, intensive *GADD45β*, and weak–medium *FoxO3a* expression levels. *GADD45β* expression levels discriminated best between sensitive and resistant cell lines.

Analyzing the impact of sorafenib on protein expression, we observed that the sensitive cell line SW480 reacts with a *pp53* and a *GADD45β* upregulation upon increasing sorafenib doses. In contrast, resistant cell lines revealed primarily intense (Caco2, SW620) and, upon sorafenib exposure, decreasing (Caco2) *GADD45β* expression levels. Similarly, both resistant cell lines decreased *pp53* levels upon exposure to increasing sorafenib doses.

### In vivo xenograft model

**Fig. 3:**
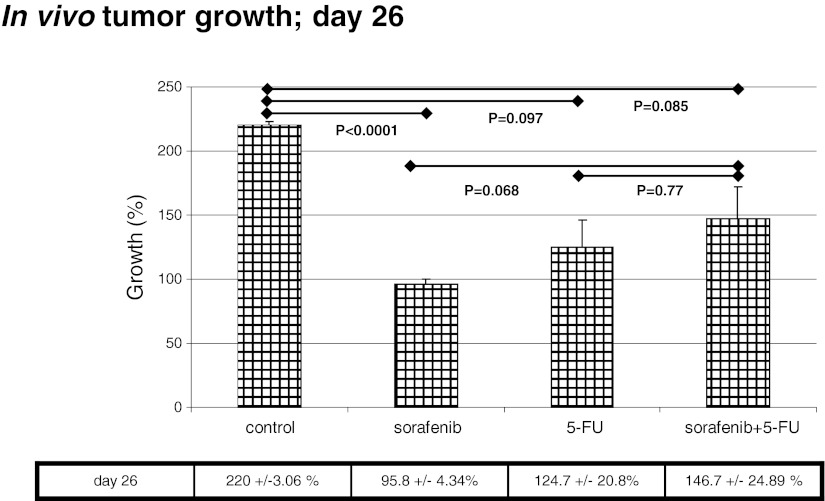
In vivo, only sorafenib monotherapy inhibited tumor growth significantly as compred to the control group. 5-FU treatment or the combination demonstrated only a nonsignificant inhibition. When all treatment groups were compared, no significant differences were observed

Ex vivo analyses of tumor size indicated that—compared to placebo control—only a sorafenib monotherapy significantly decreased tumor size (220 ± 3.06 % versus 95.8 ± 4.34 %; *P* = <0.0001), while a 5-FU monotherapy inhibited tumor growth nonsignificantly (220 ± 3.06 % versus 124 ± 20.8 %; *P* = 0.097) (Fig. 3). The 5-FU plus sorafenib combination therapy was equipotent to 5-FU monotherapy (220 ± 3.06 % versus 146 ± 24.89 %; *P* = 0.085), but inferior compared to sorafenib monotherapy (*P* = 0.068), in this explorative analysis.

#### Therapeutic effect on Ki-67 proliferation index

Analysis of the proliferation index showed that sorafenib monotherapy and 5-FU monotherapy were equally effective in reducing the proliferation index as compared to placebo. However, the combination therapy of sorafenib and 5-FU did not result in further reduction of proliferation rates.

**Fig. 4:**
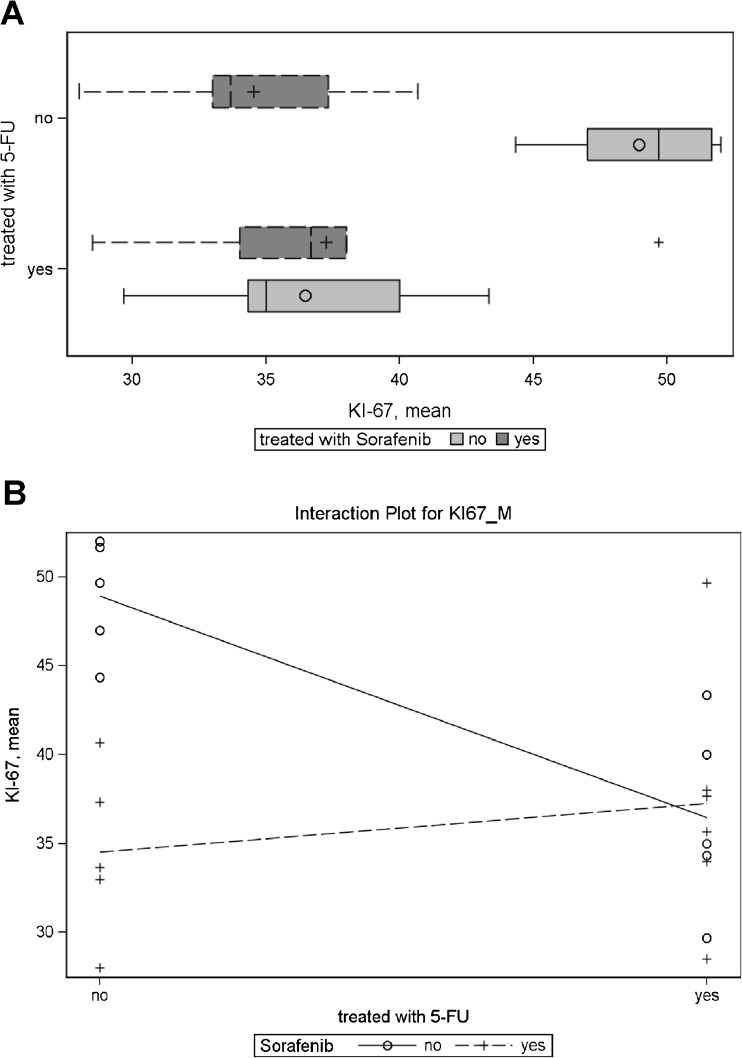
**a** Analysis of Ki-67 proliferation index in three different treated animal groups (sorafenib monotherapy versus 5-FU monotherapy versus combination therapy) show a significant reduction of proliferation in all treatment groups but no superior therapeutic effect of the combination therapy group. **b** Interaction plot for Ki-67 depicts no additive effect of combination therapy with 5-FU and sorafenib. Analysis of the expression intensity of PDGFA, PDGFRβ, and VEGFR1 in the control group and under treatment with sorafenib monotherapy, 5-FU monotherapy, and combination therapy. PDGFRβ and VEGFR1 expression intensities were significantly reduced by 5-FU and sorafenib, respectively

For Ki-67, analysis of variance demonstrated a significant effect for sorafenib (*p* = 0.0101), and for 5-FU + sorafenib interaction (*p* = 0.0049), the 5-FU main effect was borderline significant (*p* = 0.0537) as compared to the control group. On average, treatment with 5-FU lowered Ki-67 expression by 4.9 %, treatment with sorafenib on average lowered Ki-67 by 6.8 %. However, the effect was not additive: animals treated with both substances had higher Ki-67 values than animals treated with only one substance. The means for each treatment group are given in Fig. 4.

#### Therapeutic effect on cytokine expression

Specimens (*N* = 21) resected from 20 animals were included in the analysis. Each treatment group consisted of five animals, with six specimens available in the group treated with combined 5-FU and sorafenib (due to two tumors in one animal). The distribution of growth factor expression is given in Table 4. A summary of the Ki-67 measurements is given in Table 5.

**Table 4:** Summary measures for growth factor expression in treatment groups

Treated with 5-FU	Treated with sorafenib	Label	*N*	Minimum	Lower quartile	Median	Upper quartile	Maximum
No	No	VEGFR 1	5	2	2	2.5	2.5	2.5
		VEGFR 2	2	2	2	2	2	2
		VEGFR 3	5	1.5	2	2.5	3	3
		PDGFR alpha	5	0	0.5	0.5	1	1.5
		PDGFR beta	5	2	2	2	2	2
		VEGF A in cytoplasm	5	0.5	2	2.5	3	3
		VEGF A in nucleus	3	2	2	2	2	2
		PDGF A	5	0	1	1.5	1.5	2
	Yes	VEGFR 1	5	0.5	0.5	1	1	1
		VEGFR 2	5	0	0	0.5	1	1.5
		VEGFR 3	5	1.5	1.5	2	2.5	3
		PDGFR alpha	5	0	0.5	0.5	0.5	1.5
		PDGFR beta	5	0	0.5	0.5	0.5	1
		VEGF A in cytoplasm	5	1	1	1	1	2
		VEGF A in nucleus	5	0	0	0	1	2
		PDGF A	5	0	0	0	1	1.5
Yes	No	VEGFR 1	5	0.5	0.5	1	1	1.5
		VEGFR 2	5	0	0	0	0.5	1
		VEGFR 3	5	1	1.5	1.5	2	3
		PDGFR alpha	5	0	0	0.5	1.5	1.5
		PDGFR beta	5	0	0	0	1	1.5
		VEGF A in cytoplasm	5	0.5	1	1	2	2.5
		VEGF A in nucleus	5	0	0	1	2	3
		PDGF A	5	0	0	0	0	0.5
	Yes	VEGFR 1	6	1	1	1.3	1.5	1.5
		VEGFR 2	4	0	0	0.3	1	1.5
		VEGFR 3	6	1.5	2	2.3	2.5	3
		PDGFR alpha	6	0.5	0.5	1	1	1
		PDGFR beta	6	0	0	0.5	0.8	1
		VEGF A in cytoplasm	6	1	1	1	1	1.5
		VEGF A in nucleus	6	0	0	1	2	2
		PDGF A	6	0	0	0	1	1

**Table 5:** Summary of Ki-67 measurements

Treated with 5-FU	Treated with sorafenib	*N*	Mean	Standard deviation	Minimum	Lower quartile	Median	Upper quartile	Maximum
No	No	5	48.9	3.3	44.3	47	49.7	51.7	52
	Yes	5	34.5	4.8	28	33	33.7	37.3	40.7
Yes	No	5	36.5	5.3	29.7	34.3	35	40	43.3
	Yes	6	37.3	7	28.5	34	36.7	38	49.7

**Fig. 5:**
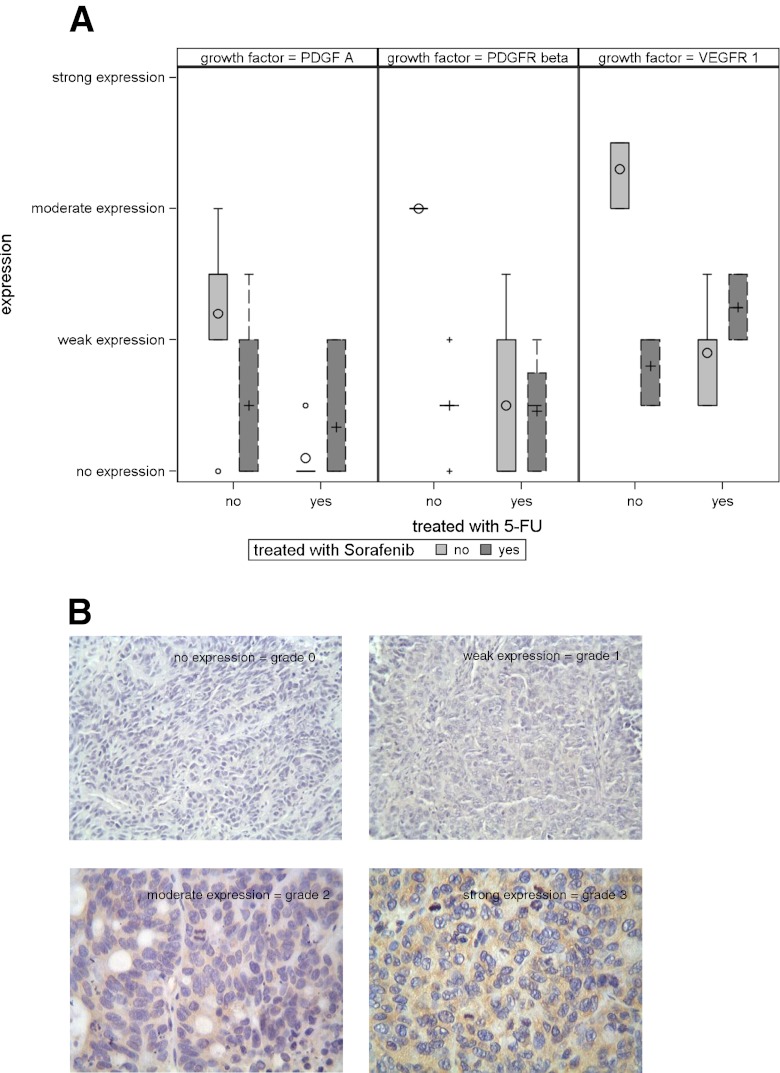
**a** Analysis of the expression intensity of PDGFA, PDGFRβ, and VEGFR1 in the control group and under treatment with sorafenib monotherapy, 5-FU monotherapy, and combination therapy. PDGFRβ and VEGFR1 expression intensities were significantly reduced by 5-FU and sorafenib, respectively. **b** Immunostaining was evaluated by three authors independently. The immunohistochemical staining was analyzed according to a scoring method as previously validated and described by Laverdiere et al. The tumors were classified into four groups based on the homogeneous staining intensity: 0, no expression; 1, weak expression; 2, moderate expression; and 3, strong expression. In the case of heterogeneous staining within the same sample, the respective higher score was chosen if more than 50 % of cells revealed a higher staining intensity. If evaluations did not agree, specimens were re-evaluated and reclassified according to the assessment given most frequently by the observers

There were substantial differences in the expression of PDGFRβ (*p* = 0.002) and VEGFR1 (*p* = 0.0002) between the control group and the different treatment groups. There was also a tendency for different expression of PDGFA (*p* = 0.0799) between different groups (Fig. 5a). Representative tissue examples are shown in Fig. 5b.

The highest expression levels of VEGFR1 were seen in the untreated control group and differed in relation to the group treated with 5-FU (*p* = 0.0079), sorafenib (*p* = 0.0079), and 5-FU + sorafenib (*p* = 0.0022). The difference among treatment groups remained within random variation.

A similar result was seen for PDGFRβ expression. The untreated control group showed the highest expression level differing from all treatment groups (*p* = 0.0079 when compared with 5-FU or sorafenib, *p* = 0.0043 when compared to 5-FU + sorafenib).

The tentative differences in PDGFA expression resulted from higher values in the untreated control group as compared to lower values in the treated groups.

The other growth factors did not show any differences beyond random variation(*p* > 0.1). The *p* values in the Kruskal–Wallis test were: PDGFRα, *p* = 0.8355; VEGFR2, *p* = 0.1020; VEGFR 3, *p* = 0.5058; cytoplasmic VEGF A, *p* = 0.2701; and nuclear VEGF A, *p* = 0.3397.

## Discussion

The approach of inhibiting RTKs with sorafenib has been successful in renal and hepatocellular cancers [17, 18]. A phase I study revealed disease stabilization in pretreated colorectal cancer patients [20]. Except of one recent study with Regorafenib, recent phase II/III studies testing other multi-tyrosine kinase inhibitors in colorectal cancer failed to show any benefit [21, 22]. So far, no molecular markers have been identified which are helpful in stratifying the patients.

We performed defined functional in vitro analyses in order to identify sorafenib-sensitive and sorafenib-resistant cell lines. While HT29 and SW480 were found to be sorafenib sensible, Caco2 was resistant and SW620 showed features of resistance. However, the mutation status of K-ras, B-Raf, PI3K, or p53 did not correlate with resistance.

Combining sorafenib with chemotherapeutic drugs used in colorectal cancer revealed an additive effect in growth inhibition and apoptosis induction in SW480 (except for oxaliplatin) and HT29 cells, whereas in Caco2 cells, apoptosis was not increased and proliferation even stimulated (5-FU or oxaliplatin). These data are in line with previous reports describing a reduced cellular uptake of oxaliplatin and generation of DNA adducts in specific colorectal cancer cells through sorafenib [23]. Thus, combination with oxaliplatin seems disadvantageous in specific settings. The effect of sorafenib on migration was marginal and of no significant importance.

Induction of apoptosis might explain the different observations made upon sorafenib exposure: While activity of caspases 6, 8, and 10 was induced in sensitive SW480 cells, it was decreased in the resistant cell lines. Furthermore, SW480 reacted with an increased activity of caspase 9. In contrast, activity of caspase 3 was decreased in Caco2 cells upon exposure to sorafenib. An induction of caspase 3 activity, as seen in prostate cancer cells, was not observed in colorectal cancer cells [24]. Our data reveal that resistance to sorafenib is associated with inhibition of specific pro-apoptotic pathways. However, sorafenib is also known to induce caspase-independent apoptosis, mediated through nuclear translocation of *AIF* [25].

We observed an inhibition of the Ras–Raf pathway (*pMEK*) in SW620 cell lines only, matching sorafenib’s function as a Raf inhibitor [25]. While sensitive cell lines revealed only a weak–absent *pAKT* expression, *AKT* expression was clearly suppressed upon exposure with increasing sorafenib doses. In contrast, the resistant cell line Caco2 did not show such *AKT* suppressive behavior. These observations match a previous report that a constitutively active *AKT* protects cells against sorafenib/bortezomib-induced apoptosis [26, 27].

Sorafenib-sensitive cells lines were defined by almost absent *pAKT*, medium–strong *FoxO3a*, and hint *GADD45β* levels. The tumor suppressor *FoxO3* belongs to a subclass of the forkhead transcription factors, being inhibited by activation of the PI3K pathway. Downregulation of *FoxO3* is thus considered a consequence of *pAKT* activity.

In contrast, resistant cell lines showed medium *pAKT*, weak *FoxO3a*, and very intense *GADD45β* levels. *GADD45β* expression levels discriminated best between sensitive and resistant cell lines. *GADD45* is a stress sensor modulating the response of cells to genotoxic or oxidative stress [28–30]. In specific colon cancer cells, *GADD45β* over-expression was linked to protection from platin induced death, matching our observations [31]. Being an apoptosis modulator, activation of *GADD45β* prevents the propagation of damaged cells, causing an arrest in cell growth and apoptosis after exposure to toxins [32]. This regulation seems intact in SW480 cells but reversed in resistant cells; *GADD45ß* was downregulated in Caco2 upon sorafenib treatment, going along with a sorafenib-mediated inhibition of caspases 6, 8, and 10. As a downstream effector of *p53*, *GADD45β* was confirmed to be specifically downregulated in HCC, which was associated to the extent of *p53* mutation [33]. We observed a *pp53* and a *GADD45β* upregulation in some sensitive cell line (SW480) upon exposure to sorafenib. In contrast, resistant cell lines showed primarily high (Caco2, SW620) and, upon sorafenib exposure, decreasing (Caco2) *GADD45β* and *pp53* levels. These data are in line with observations in HCC, in which *GADD45β* induction by sorafenib occurred only in sensitive hepatocellular carcinoma cell lines, independent of the *Raf/MEK/ERK* signaling pathway [34].These findings confirm our definition of sensitive cell lines, in which sorafenib induces apoptosis and inhibits proliferation.

In vivo, Wilhelm and colleagues described a potent growth inhibition of HT29 xenografts at sorafenib doses of 7.5 mg/kg. We studied four different groups in vivo: placebo, 5-FU, sorafenib, and 5-FU + sorafenib. 5-FU was chosen, being the backbone of most chemotherapeutic protocols in colorectal cancer. Sorafenib was applied at 5 mg/kg, matching 400 mg/day as used in combination therapies [19, 20].

Interestingly, we observed that a sorafenib monotherapy was at least equally effective as the 5-FU monotherapy or as the combination therapy and even tended to inhibit in vivo tumor growth somewhat better than the combination therapy.

The proliferation index was significantly reduced in all treatment groups as compared to the control group but displayed similar results for mono-agent therapy and the combination therapy. Since only small numbers were analyzed, a possibility exists that larger treatment groups might demonstrate even more distinct differences. However, we clearly demonstrate that combination of sorafenib and chemotherapy did not result in any additive effects. In contrast, it seems that treatment effects are partially cancelled when 5-FU and sorafenib are applied simultaneously.

Expression rates of receptor tyrosine kinases VEGFR1 and PDGFRβ as well as of the ligand PDGFA were decreased by all treatment regimens used. However, no significant differences were detected between treatment groups.

Inhibition of receptor tyrosine kinases through sorafenib could potentially lead to a selection of low target expressing tumor cells. Combination regimens of sorafenib and 5-FU might reduce sorafenib target expression leading to a similar proliferation effect as under 5-FU monotherapy. However, the adverse events in humans might rather be additive. Our results indicate that there is no additive effect in combination of these two treatment mechanisms and that combination might only add adverse events. Therefore, in future studies preferentially sorafenib monotherapy versus sequential treatment regimens (inductiontherapy via chemotherapy–maintenance via sorafenib) should be explored.

## Conclusion

Diverse tyrosine kinase inhibitors have failed in colorectal cancer. However, sorafenib still seems promising in distinct settings, if applied as monotherapy. In our human colon cancer xenograft model, it seems that treatment effects are partially cancelled when 5-FU and sorafenib are applied simultaneously. However, monotherapy with sorafenib seems to be sufficient for tumor control in our human colon cancer xenograft model, especially considering the application advantage and toxicity profile.
